# Supplementary material for: Network-informed discovery of multidrug combinations for ERα+/HER2-/PI3Kα-mutant breast cancer
Source: Cell Mol Life Sci. 2023 Mar 3;80(3):80. doi: 10.1007/s00018-023-04730-x (PMC10032341; doi:10.1007/s00018-023-04730-x)
Supplement: Supplementary file 1 — Supplementary Information (PDF 8100 KB) [file 18_2023_4730_MOESM1_ESM.pdf]

Supplementary Information

Network-informed discovery of multidrug combinations for ERα+/HER2-/PI3Kα-mutant breast cancer

Hany et al.

This file includes:

Supplementary Discussion
Description of Tables S1 to S3
Tables S4 and S5
Description of the data files Data S1 to S5
Figures S1 to S10 with corresponding legends

Supplementary Discussion

Why is there no benefit of adding the PARP1 inhibitor talazoparib in xenograft experiments?

As mentioned in the main text, we discovered a promising 4-drug combination referred to as AUTTala, of which talazoparib is one component. While the discovery itself and cell culture experiments in general revealed a clear benefit of adding talazoparib, we failed to see it in mouse xenograft experiments. This might be due to several factors: (i) The fact that MCF7 cells are highly sensitive to tamoxifen would limit the benefit of talazoparib that was obvious in cases of long-term treatment of tamoxifen-tolerant or resistant cells (Fig. 6F, G); (ii) the dose and pace of administration of talazoparib may need further optimization since talazoparib showed high toxicity at the selected doses during the initial stages of the study; despite the fact that we further reduced the dose of talazoparib as part of the combination AUTTala by 20x, its dose might still not be optimal; (iii) talazoparib is mainly excreted in urine [1], which might be incompatible with the use of estradiol pellets, which in the mouse were shown to cause urine retention, obstruction, cystitis, and hydronephrosis [2–5]. Although urine retention was prevented by manual bladder expression, hypothetically there could still be some impairment of kidney function, which would lead to increased plasma concentrations of talazoparib, and hence, high toxicity. Based on the available *in vitro* and clinical data for talazoparib, an inhibitory drug-drug interaction among alpelisib, tamoxifen, and talazoparib is less likely, but if anything, it might be potentiating, as discussed in the next chapter.

Drug-drug interactions

Tamoxifen is highly metabolized by liver enzymes, including the cytochrome P450s CYP2C9, CYP2D6, and CYP3A4, to its 30-100x more active metabolites. The major metabolic pathways of tamoxifen are the N-de-methylation (around 92%) and 4-hydroxylation (around 7%), which yield N-desmethyltamoxifen and OHT as primary active metabolites, respectively [6, 7]. These metabolites undergo a second conversion to another highly potent form, 4-hydroxy-N-desmethyltamoxifen (known as endoxifen), which represents the most abundant metabolite [6, 7]. Alpelisib was shown with *in vitro* assays to induce CYP2C9 and CYP3A4 [8], which are involved in the conversion of tamoxifen to OHT and N-desmethyltamoxifen. A minor proportion of alpelisib is metabolized by the enzyme CYP3A4 [9], which can be affected by tamoxifen co-administration. Since tamoxifen can inhibit the activity of this enzyme, the alpelisib concentration might be raised. Moreover, tamoxifen, alpelisib, and talazoparib are substrates of the efflux pumps p-glycoprotein [8, 10, 11] and BCRP [8, 12, 13] at varying affinities, which might allow competitive interactions. Efflux pumps are widely present in the cells of multiple organs, such as the small intestines, tubules of the kidneys, and the liver, in addition to the cancer cells themselves, which can render them drug resistant. Inhibition of efflux pumps by one or more of the drugs might increase the plasma concentrations of the others and/or itself and increase the intracellular drug concentration in cancer cells. Therefore, monitoring of therapeutic doses and plasma concentrations might be recommended.

References for Supplementary Discussion

1. Hoy SM (2018) Talazoparib: First global approval. Drugs 78:1939–1946. DOI:10.1007/S40265-018-1026-Z
2. Buhl AE, Yuan YD, Cornette JC, Frielink RD, Knight KA, Ruppel PL & Kimball FA (1985) Steroid-induced urogenital tract changes and urine retention in laboratory rodents. J Urol 134:1262–1267. DOI:10.1016/S0022-5347(17)47708-1
3. Walker SE, McMurray RW, Besch-Williford CL & Keisler DH (1992) Premature death with bladder outlet obstruction and hyperprolactinemia in New Zealand black X New Zealand white mice treated with ethinyl estradiol and 17 beta-estradiol. Arthritis Rheum 35:1387–1392. DOI:10.1002/ART.1780351123
4. Pearse G, Frith J, Randall KJ & Klinowska T (2009) Urinary retention and cystitis associated with subcutaneous estradiol pellets in female nude mice. Toxicol Pathol 37:227–234. DOI:10.1177/0192623308329281
5. Gakhar G, Wight-Carter M, Andrews G, Olson S & Nguyen TA (2009) Hydronephrosis and urine retention in estrogen-implanted athymic nude mice. Vet Pathol 46:505–508. DOI:10.1354/VP.08-VP-0180-N-BC
6. Klein DJ, Thorn CF, Desta Z, Flockhart DA, Altman RB & Klein TE (2013) PharmGKB summary. Pharmacogenet Genomics 23:643–647. DOI:10.1097/FPC.0b013e3283656bc1
7. Lien EA, Solheim E, Lea OA, Lundgren S, Kvinnsland S & Ueland PM (1989) Distribution of 4-hydroxy-N-desmethyltamoxifen and other tamoxifen metabolites in human biological fluids during tamoxifen treatment. Cancer Res 49:2175–2183
8. European Medicines Agency (CHMP) (2020) Piqray, assessment report.
9. James A, Blumenstein L, Glaenzel U, Jin Y, Demailly A, Jakab A, Hansen R, Hazell K, Mehta A, Trandafir L, *et al* (2015) Absorption, distribution, metabolism, and excretion of [(14)C]BYL719 (alpelisib) in healthy male volunteers. Cancer Chemother Pharmacol 76:751–760. DOI:10.1007/S00280-015-2842-4
10. Callaghan R & Higgins CF (1995) Interaction of tamoxifen with the multidrug resistance P-glycoprotein. Br J Cancer 71:294–299. DOI:10.1038/bjc.1995.59
11. Elmeliegy M, Láng I, Smolyarchuk EA, Chung CH, Plotka A, Shi H & Wang D (2020) Evaluation of the effect of P-glycoprotein inhibition and induction on talazoparib disposition in patients with advanced solid tumours. Br J Clin Pharmacol 86:771–778. DOI:10.1111/BCP.14178
12. Guney Eskiler G, Cecener G, Egeli U & Tunca B (2020) Talazoparib nanoparticles for overcoming multidrug resistance in triple-negative breast cancer. J Cell Physiol 235:6230–6245. DOI:10.1002/JCP.29552
13. Selever J, Gu G, Lewis MT, Beyer A, Herynk MH, Covington KR, Tsimelzon A, Dontu G, Provost P, Di Pietro A, *et al* (2011) Dicer-mediated upregulation of BCRP confers tamoxifen resistance in human breast cancer cells. Clin Cancer Res 17:6510–6521. DOI:10.1158/1078-0432.CCR-11-1403

Description of Tables S1 to S3 (available as separate files):

Table S1 Details and doses of the anticancer drugs used throughout the study. The Microsoft Excel workbook contains information about the commercial source of the drugs, biological targets, drug development phase, physical properties, stock concentrations, and doses used for the TGMO-based screens, molecular assays, and xenograft experiments.

Table S2 A list of predicted synergistic 2-, 3-, and 4- drug combinations from “Round 2” of the TGMO-based screen. The Microsoft Excel workbook contains the list of candidate drug combinations for MCF7, MCF7-V, and MCF7/LCC2 cells, and their potential safety profile in MCF10A cells. Combinations of potential therapeutic relevance were selected for “Round 3” assays.

Table S3 Status of *PIK3CA* gene mutations in different breast cancer cell lines. The Microsoft Excel workbook contains the status of the tested mutations of the *PIK3CA* gene in exons 9 and 20 in the different breast cancer cell lines used in the study.

Table S4 List of antibodies.

Antigen and/or antibody	Supplier	Catalog no.	Host	Clonality	Dilution
Caspase 3	Cell Signaling Technology	9662	Rabbit	Polyclonal	1:1,000
Caspase 7, clone: (C7)	Cell Signaling Technology	9494	Mouse	Monoclonal	1:1,000
NFκB2 p100/p52	Cell Signaling Technology	4882	Rabbit	Polyclonal	1:1,000
Phospho-Thr145 of p21	Abbkine	ABP50378	Rabbit	Polyclonal	1:1,000
p21 Cip1, clone: [GT1032]	GeneTex	GTX629543	Mouse	Monoclonal	1:1,000 for western blot and 1:500 for immuno-fluorescence
Rad51, clone [N1C2]	GeneTex	GTX100469	Rabbit	Polyclonal	1:1,000
Lamin B1	GeneTex	GTX103292	Rabbit	Polyclonal	1:1,000
Phospho-Ser139 of histone γ-H2AX, clone: [GT1021]	GeneTex	GTX628996	Mouse	Monoclonal	1:2,000 for western blot and immuno-fluorescence
PARP (cleaved Asp214)	GeneTex	GTX132329	Rabbit	Polyclonal	1:1,000
ERK 2, clone: (C-14)	Santa Cruz Biotechnology	Sc-154	Rabbit	Polyclonal	1:200
Phospho-Tyr 204 of ERK, clone: (E-4)	Santa Cruz Biotechnology	Sc-7383	Mouse	Monoclonal	1:1,000
Phospho-Tyrosine (P-Tyr-100)	Cell Signaling Technology	9411	Mouse	Monoclonal	1:2,000
a-tubulin, clone (DM1A)	Merck Millipore	CP06	Mouse	Monoclonal	1:5,000

GAPDH, clone (6C5)	Abcam	ab8245	Mouse	Monoclonal	1:30,000
Phospho-S311 of NFκB p65	Santa Cruz	sc-135769	Mouse	Monoclonal	1:1,000
Mouse IgG (H+L), HRP secondary antibody	Invitrogen	31430	Goat	Polyclonal	1:10,000
Rabbit IgG (H+L), HRP secondary antibody	Invitrogen	31460	Goat	Polyclonal	1:10,000
Mouse IgG (H+L), Alexa Fluor 488	Invitrogen	A-11017	Goat	Polyclonal	1:5,000
Mouse IgG (H+L), Alexa Fluor 546	Invitrogen	A-11030	Goat	Polyclonal	1:1,000

Table S5 List of specific primer sequences used for real-time RT-qPCR

**Gene**	**Forward 5'-3'**	**Reverse 5'-3'**
VEGF	CAGAAGGAGGAGGGCAGAATC	GTCCACCAGGGTCTCGATTG
CDKN1A	AGGTGGACCTGGAGACTCTCAG	TCCTCTTGGAGAAGATCAGCCG
CDKN2A	CTCGTGCTGATGCTACTGAGGA	GGTCGGCGCAGTTGGGCTCC
CDKN1B	ATAAGGAAGCGACCTGCAACCG	TTCTTGGGCGTCTGCTCCACAG
TP53	TAGTGTGGTGGTGCCCTATGA	ACACGCACCTCAAAGCTGTTC
β-ACTIN	CATGTACGTTGCTATCCAGGC	CTCCTTAATGTCACGCACGAT

Description of the Data files S1 to S5 (available as separate files):

Data S1 ERα PPI network. The Cytoscape file contains the network of primary PPIs of ERα with 322 protein interactors and secondary PPIs among them. Based on the analysis of network centrality measures, the top 50 interactors are highlighted in green.

Data S2 List of ERα interactors. The Microsoft Excel workbook contains the list of 322 ERα interactors that were given the feature "DP". The file was imported to Cytoscape to be integrated into the network of the human interactome.

Data S3 Analysis of ERα PPI network centrality measures. The Microsoft Excel workbook of the results of the network analysis done by Cytoscape on the ERα PPI. Results include coefficients for network centrality parameters, such as "Number of directed edges", "Radiality", "Stress", and others.

Data S4 Genes co-expressed with ERα in ERα+ breast cancer datasets. The Microsoft Excel workbook contains the co-expression coefficients obtained from datasets available from the online databases Oncomine (www.oncomine.org) and GOBO (http://co.bmc.lu.se/gobo).

Data S5 Integrated network of SL between interactors of ERα, co-expressed, and frequently mutated genes in ERα+ breast cancer. The Cytoscape file contains two networks; one represents SL between genes in breast cancer, and another network derived from the first one that contains only those genes that are ERα interactors, co-expressed, or frequently mutated genes in ERα+ breast cancer.

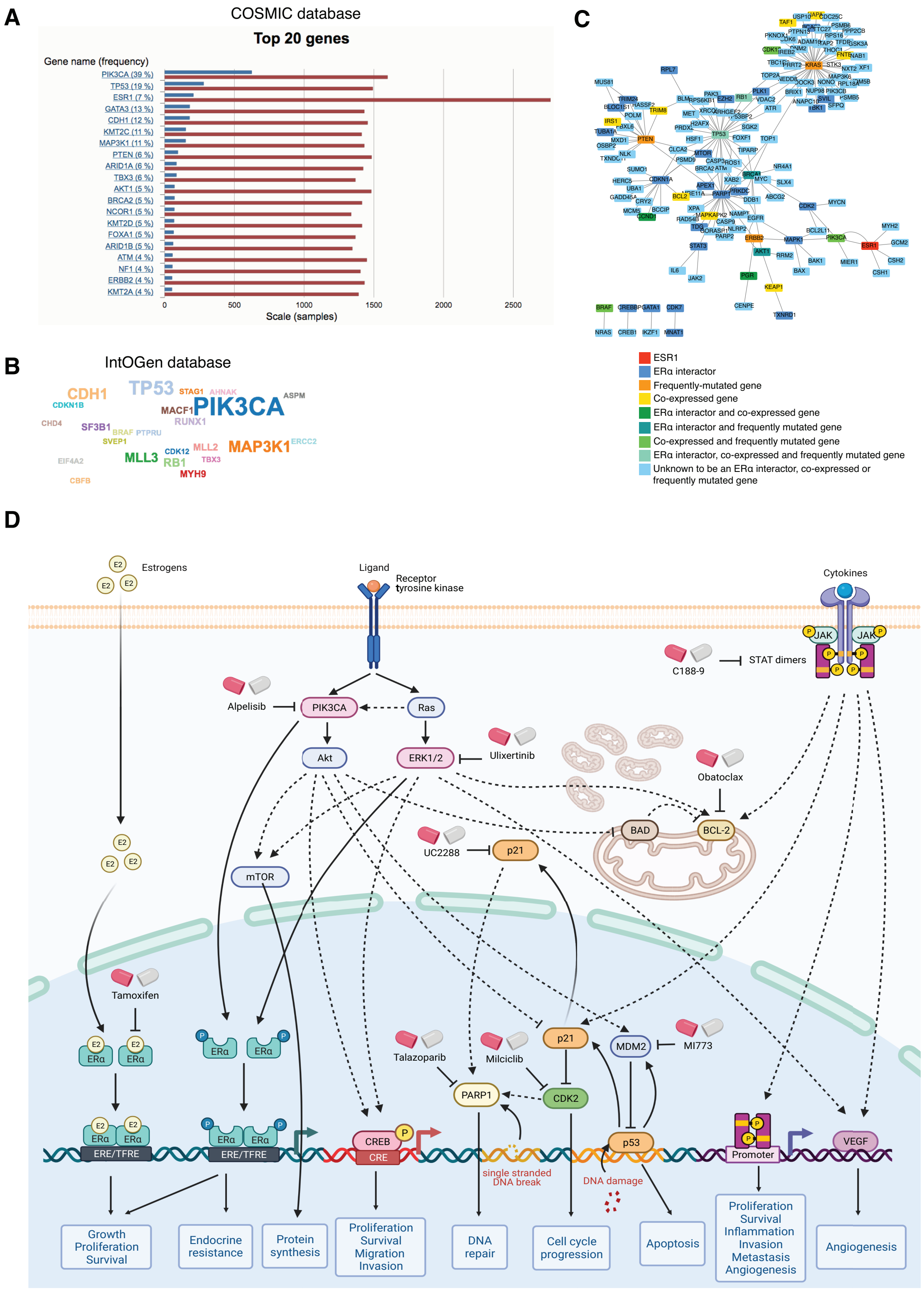

Fig. S1 Frequently mutated genes and SL network in ERα+ breast cancer, and crosstalk between potential drug targets. **(A,B)** Raw data of the most frequently mutated genes in ERα+ breast cancer obtained from the COSMIC (panel A) and IntOGen databases (panel B). In panel A, blue bars represent the number of samples with mutations and the red bars are the total number of samples analyzed. **(C)** Network

of SL relationships in breast cancer. ERα interactors, genes co-expressed with ERα, and frequently mutated genes in ERα+ breast cancer are highlighted with color codes. Nodes represent genes and edges represent SL relationships between the connected genes. **(D)** Schematic illustration of relevant signaling pathways and the network of signaling crosstalk between them. Solid lines indicate a direct effect, whereas dotted lines indicate an indirect effect. The 9 drugs selected for “round 1” of the TGMO-based screen and their primary targets are included in the scheme. The scheme was created with Biorender.com based on information from the literature and the KEGG PATHWAY database.

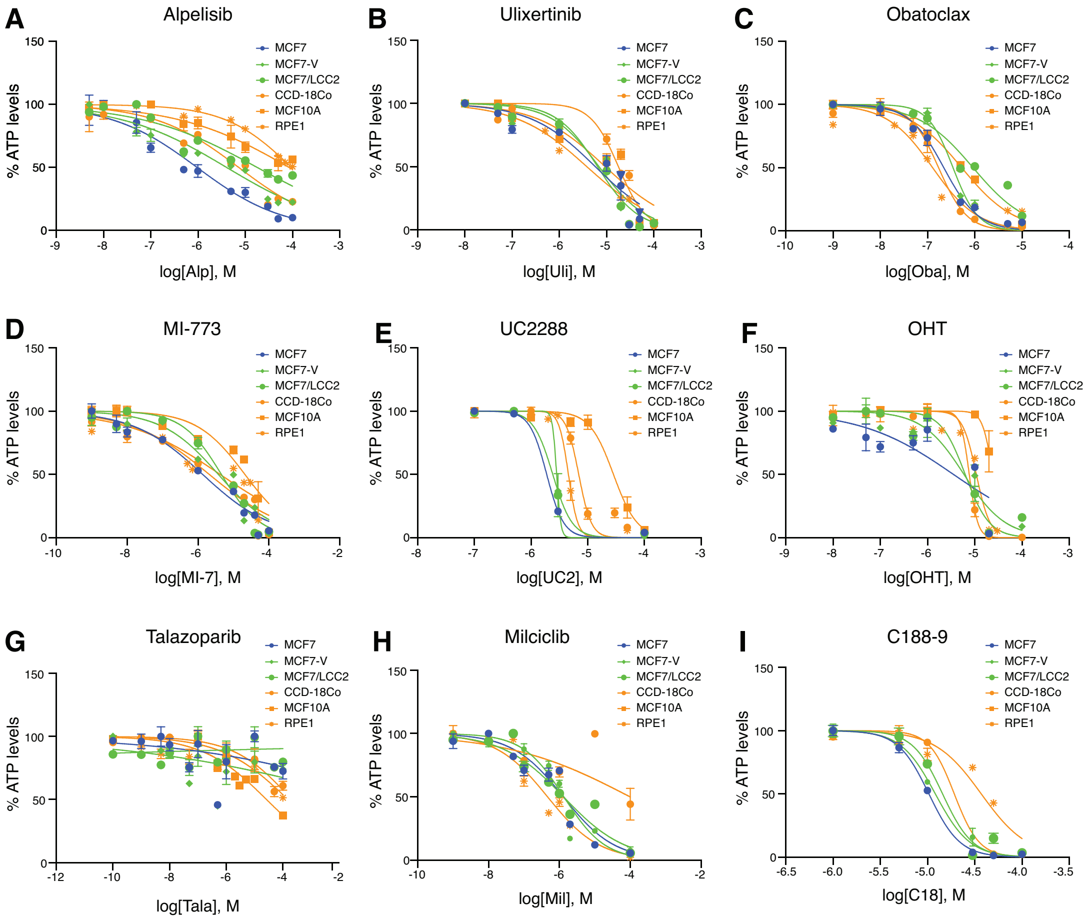

Fig. S2 Dose-response curves of the selected 9 drugs in cancer and non-cancer cell lines. **(A-I)** Dose-response curves of the 9 selected drugs for “round 1” of the TGMO-based screen. The indicated cancer (blue and green) and non-cancer (orange) cell lines were treated with increasing doses of each drug and % ATP levels were calculated as the response. For each curve, a control group treated with vehicle was set to 100%. Data are represented as means ± SD (n = at least 3 independent samples).

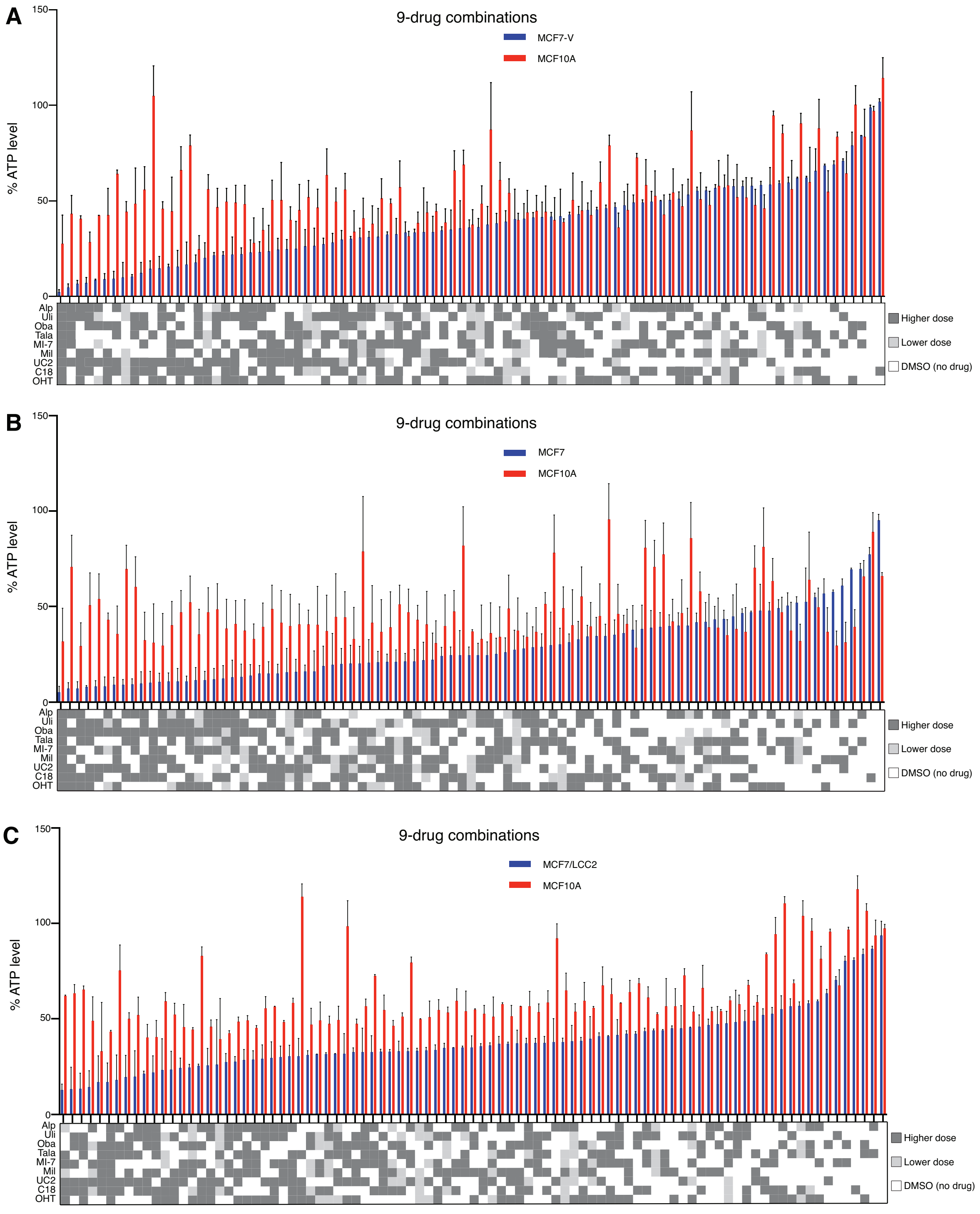

Fig. S3 Results of "Round 1" of the *in vitro* TGMO-based screen. **(A-C)** Bar graphs of the % ATP levels of the breast cancer cell lines (blue) MCF7-V (panel A), MCF7 (panel B), and MCF7/LCC2 (panel C) treated with alpelisib (Alp), ulixertinib (Uli), obatoclax (Oba), talazoparib (Tala), MI-778 (MI-7), milciclib (Mil), UC2288 (UC2), C188-9 (C18), and OHT, tested alone or in combinations. Red bars show results obtained with the non-cancerous MCF10A cells. The data are presented as means ± SEM of 3 independent experiments, each with 3 independent replicates. The matrices below the graphs represent the scheme of the combinatorial design, in addition to the vehicle

(DMSO) control in white, each drug was given at two dose levels, higher dose (dark gray) and lower dose (light gray). Values of the DMSO only controls were set to 100%.

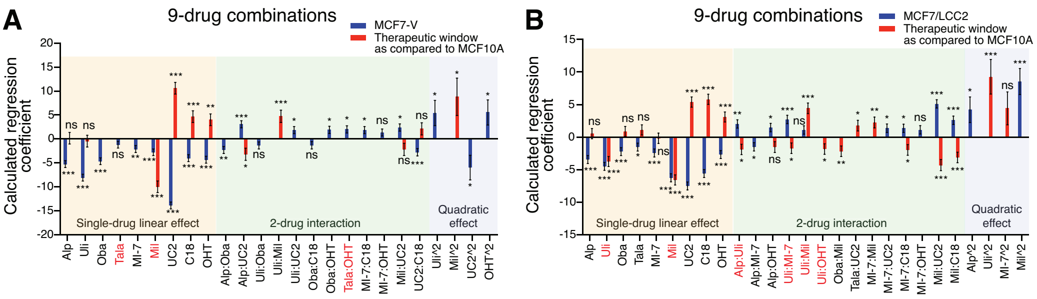

Fig. S4 Regression modeling of the results of “Round 1” of the *in vitro* TGMO-based screen. **(A,B)** Estimated regression coefficients obtained by second-order linear regression of the calculated % ATP levels from “round 1” in MCF7-V (panel A) and MCF7/LCC2 (panel B) cells. Blue bars indicate regression coefficients of cancer cells, whereas red bars are show the TW as compared to MCF10A cells. Drugs highlighted in red in the x-axis labels represent drugs or combinations with unfavorable efficacy or toxicity profiles, which were therefore eliminated from “round 2”. Statistical significance is shown as * for $p \leq 0.05$, ** for $p \leq 0.01$, *** for $p \leq 0.001$, and “ns” for non-significant results. Data are represented as means ± standard error of the means (SEM) of 3 independent experiments, each containing 3 independent replicates.

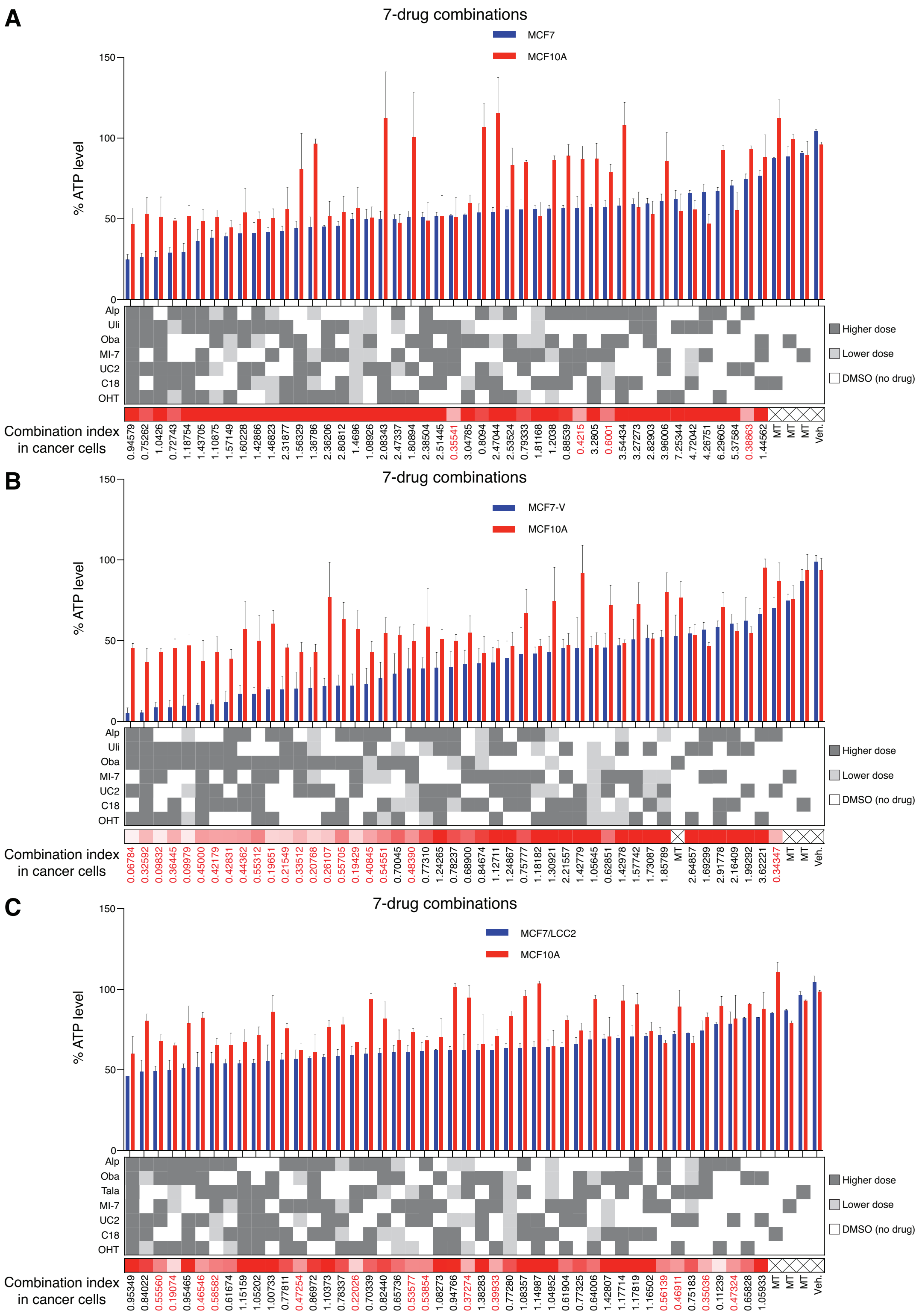
A
7-drug combinations
MCF7
MCF10A
% ATP level
150
100
50
0
Alp
Uli
Oba
MI-7
UC2
C18
OHT
Higher dose
Lower dose
DMSO (no drug)
Combination index in cancer cells
0.94579 0.75262 1.0426 0.72743 1.18754 1.43705 1.10875 1.57149 1.60228 1.42866 1.46823 2.31877 1.56329 1.36786 2.36206 2.80812 1.4696 1.08926 2.08343 2.47337 1.80894 2.38504 2.51445 0.35541 3.04785 0.8094 2.47044 2.53524 0.79333 1.81168 1.2038 0.88539 0.4215 3.2805 0.6001 3.54434 3.27273 2.82903 3.96006 7.25344 4.72042 4.26751 6.29605 5.37584 0.38863 1.44562 MT MT MT Veh.
B
7-drug combinations
MCF7-V
MCF10A
% ATP level
150
100
50
0
Alp
Uli
Oba
MI-7
UC2
C18
OHT
Higher dose
Lower dose
DMSO (no drug)
Combination index in cancer cells
0.06784 0.32592 0.09832 0.36445 0.09979 0.45000 0.42179 0.42831 0.44362 0.55312 0.19651 0.21549 0.33512 0.20768 0.26107 0.55705 0.19429 0.40845 0.54551 0.70045 0.48390 0.77310 1.24265 0.78237 0.68900 0.84674 1.12711 1.24867 0.75777 1.18182 1.30921 2.21557 1.42779 1.05645 0.62851 1.42978 1.57742 1.73087 1.85789 MT 2.64857 1.69299 2.91778 2.16409 1.99292 3.62221 0.34347 MT Veh.
C
7-drug combinations
MCF7/LCC2
MCF10A
% ATP level
150
100
50
0
Alp
Oba
Tala
MI-7
UC2
C18
OHT
Higher dose
Lower dose
DMSO (no drug)
Combination index in cancer cells
0.95349 0.84022 0.55560 0.19074 0.95465 0.46546 0.58582 0.61674 1.15159 1.05202 1.00733 0.77811 0.47254 0.86972 1.10373 0.78337 0.22026 0.70339 0.82440 0.65736 0.53577 0.53854 1.08273 0.94766 0.37274 1.38283 0.39933 0.77280 1.08357 1.14987 1.04952 0.61904 0.77325 0.64006 1.42807 1.17714 1.17819 1.16502 0.56139 0.46911 0.75183 0.35036 0.11239 0.47324 0.65828 1.05933 MT MT MT Veh.

Fig. S5 "Round 2" of the *in vitro* TGMO-based screen. **(A-C)** Bar graphs of the % ATP levels of the breast cancer cell lines (blue) MCF7 (panel A), MCF7-V (panel B), and MCF7/LCC2 (panel C) treated with alpelisib (Alp), ulixertinib (Uli) (panels A and B), obatoclax (Oba), talazoparib (Tala) (panel C), MI-778 (MI-7), UC2288 (UC2), C188-9 (C18), and OHT, tested alone or in combinations. Red bars show results obtained with MCF10A cells. Note that the same values obtained with MCF10A cells were used in all three panels (A-C) for comparison. The data are presented as means ± SEM of 3 independent experiments, each with 3 independent replicates. The matrices below the graphs represent the scheme of the combinatorial design, in addition to the vehicle (DMSO) control in white, each drug was given at two dose levels, higher dose (dark gray) and lower dose (light gray). Values of the DMSO only controls were set to 100%. CIs calculated by CompuSyn are illustrated below the combinatorial matrices as a red color gradient. Low CI < 0.8 (light red) indicates synergistic combinations, whereas high CI > 1 (dark red) indicates antagonistic combinations. MT, monotherapy; Veh., DMSO control.

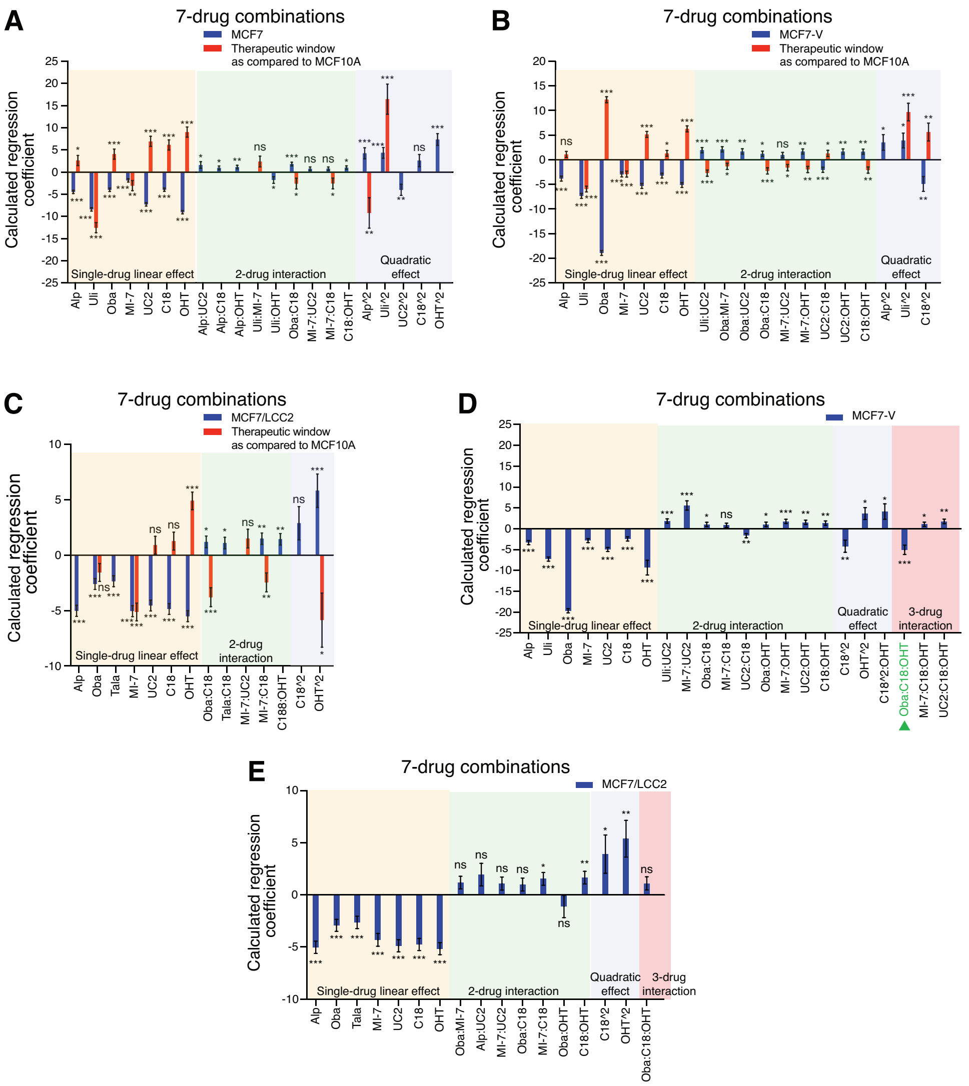

Fig. S6 Regression modeling of the results of "Round 2" of the *in vitro* TGMO-based screen. **(A-E)** Estimated regression coefficients obtained by second-order linear regression (panels A to C) or third-order linear regression (panels D and E) of the calculated % ATP levels from "round 2" in MCF7 (panel A), MCF7-V (panels B and D), and MCF7/LCC2 (panels C and E) cells. Blue bars indicate regression coefficients of cancer cells (panels A to E), whereas red bars show the TW as compared to MCF10A cells (panels A to C). For panel D, combinations highlighted in green in the x-axis labels have favorable efficacy profiles. Statistical significance is shown as * for $p \leq 0.05$, ** for $p \leq 0.01$, *** for $p \leq 0.001$, and "ns" for non-significant results. Data are represented as means ± SEM of 3 independent experiments, each with 3 independent replicates.

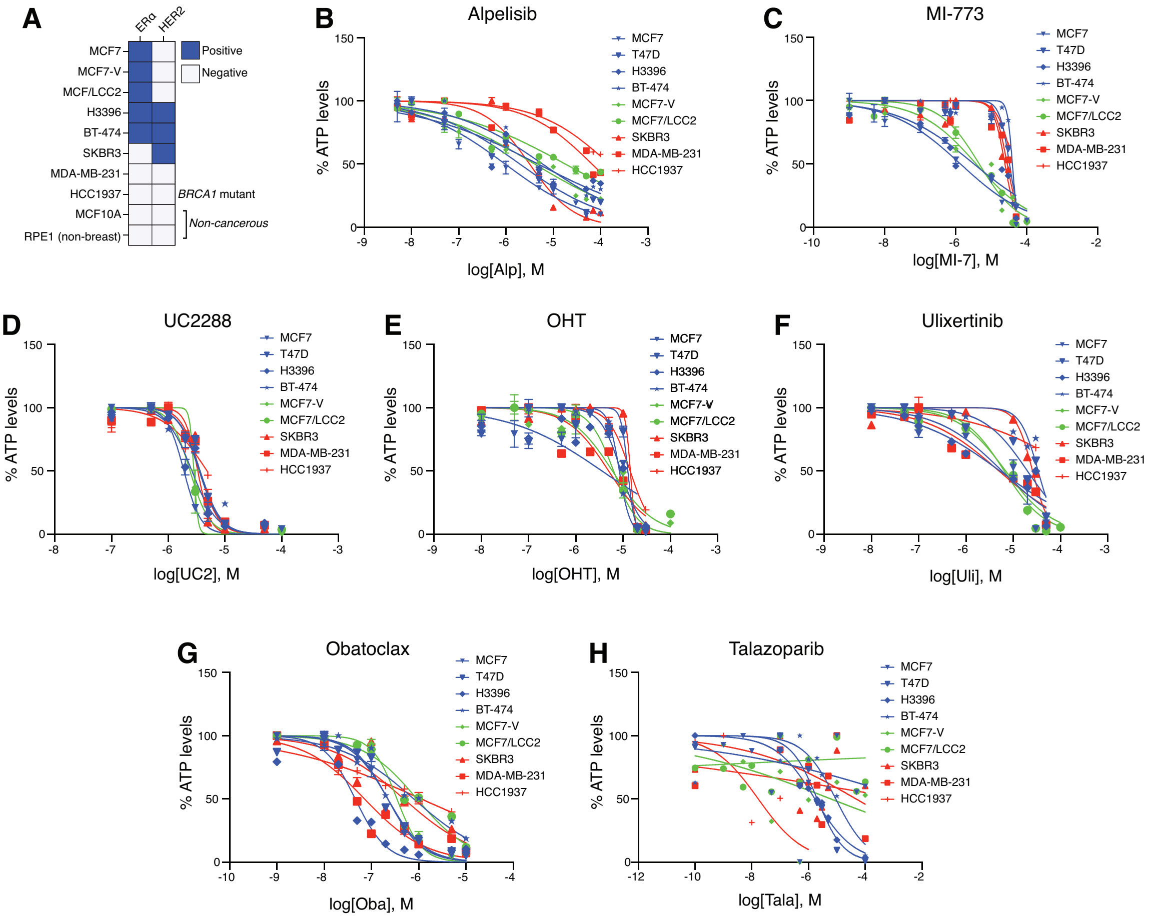

Fig. S7 Specificity of the selected 7 drugs from "Round 2" in cancer cell lines representative of the different molecular subtypes of breast cancer. **(A)** A scheme of the molecular subtypes of the cell lines used in "round 3" of the combinatorial screen based on ERα and HER2 expression. **(B-H)** Dose-response curves of the 7 selected drugs from "round 2" to be tested in different combinations in "round 3". The indicated breast cancer cell lines include ERα+ (blue), ERα+ but tamoxifen-tolerant or -resistant (green), and ERα- (red) cell lines. Cells were treated with increasing doses of each drug and % ATP levels were calculated as the response. For each curve, a control group treated with vehicle was set to 100%. Data are represented as means ± SD (n = at least 3 independent samples). Note that the same data (curves) obtained with MCF7, MCF7-V, and MCF7/LCC2 cells were used as in Fig. S2, and plotted here for comparison.

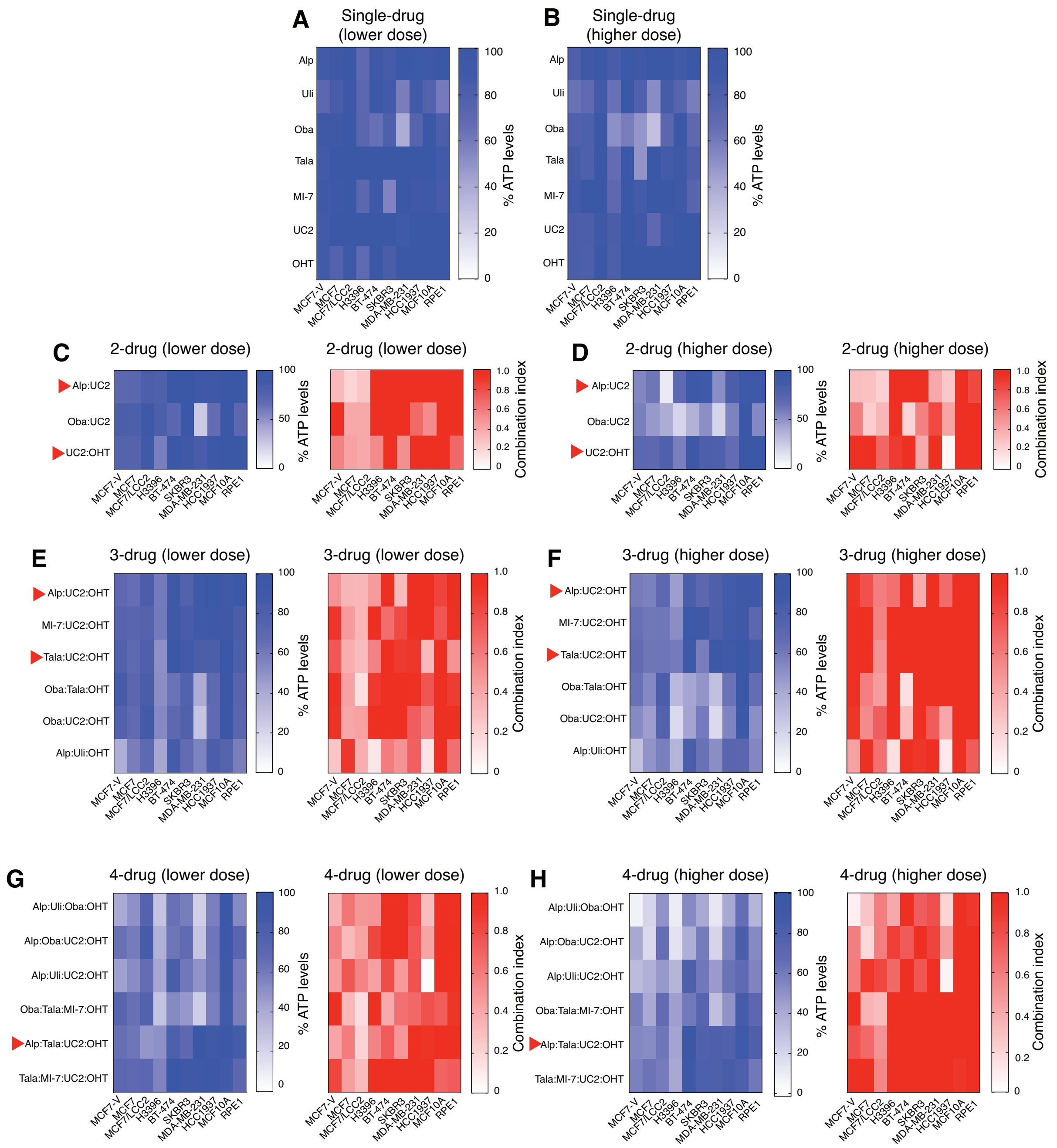

Fig. S8 "Round 3" of the *in vitro* TGMO-based screen. **(A,B)** Heatmaps of the % ATP levels (blue gradient) of the breast cancer cell lines treated with alpelisib (Alp), ulixertinib (Uli), obatoclax (Oba), talazoparib (Tala), MI-778 (MI-7), UC2288 (UC2), and OHT, tested alone at two concentrations indicated as "lower dose" (panel A) and "higher dose" (panel B). Low % ATP levels (light blue) indicates high efficacy and high % ATP levels (dark blue) indicates low efficacy. The data are presented as means of 3 independent experiments. Values of DMSO control were set to 100%. **(C-H)** Heatmaps of the % ATP levels (left side of the panel) of the predicted 2-drug (panels C and D), 3-drug (panels E and F), and 4-drug (panels G and H) combinations from "round 2". CIs calculated by CompuSyn are illustrated in the heatmap on the right of each panel (red gradient). Low CI < 0.8 (light red) indicates synergistic combinations,

whereas high CI > 1 (dark red) indicates antagonistic combinations. Combinations were tested at lower (panels C, E, and G) and higher dose levels (panels D, F, and H). Red arrows indicate combinations with higher efficacy and synergy in ERα+ breast cancer cell lines and less toxicity in non-cancerous cells.

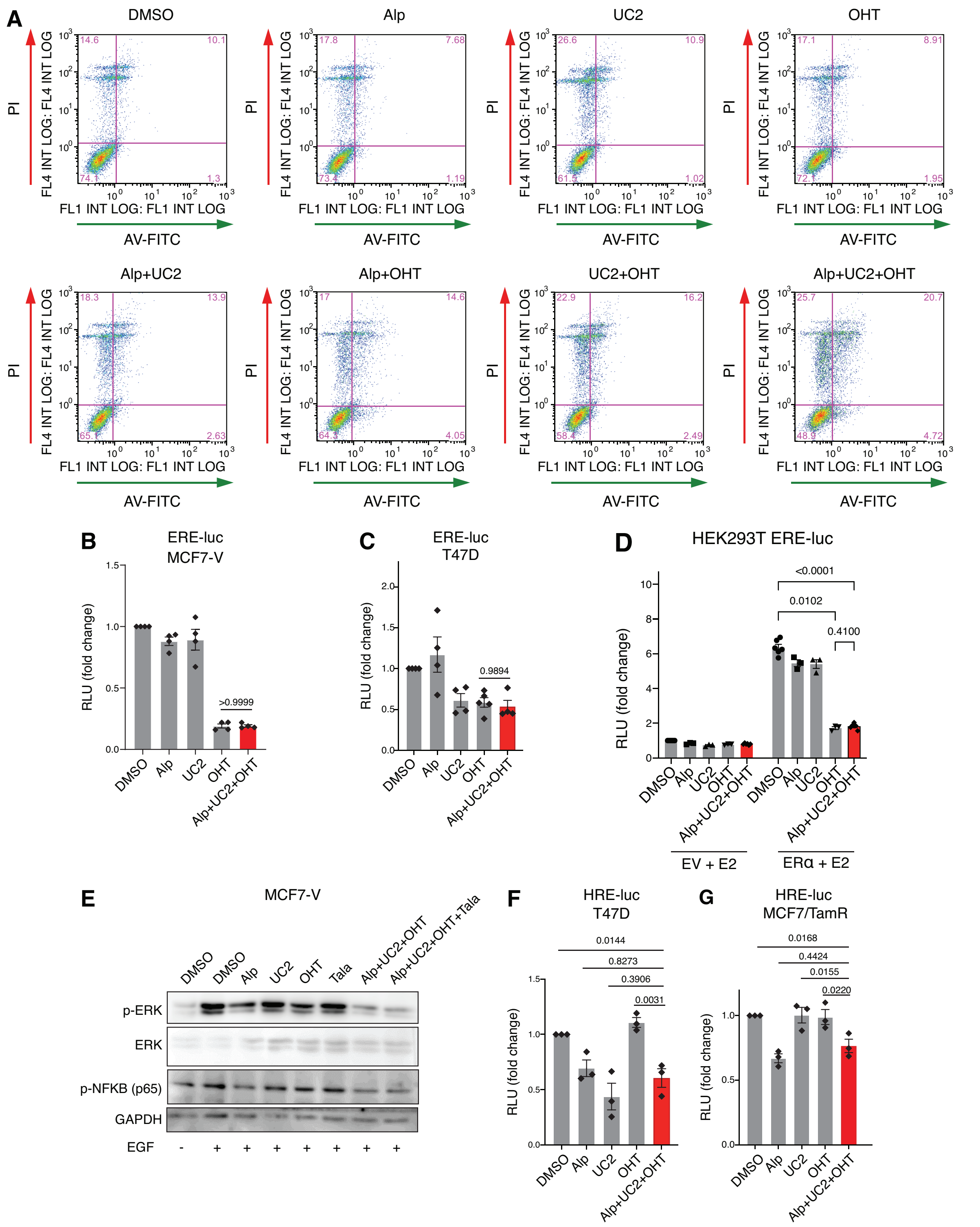

Fig. S9 FACS analysis of PI/annexin V staining and evaluation of the effects of AUT on ERα, ERK, NFκB, and HIF1α activities. **(A)** Representative dot plots of the flow cytometric analysis of MCF7-V cells double stained with PI/annexin V-FITC (AV-FITC). Cells were treated with alpelisib (Alp), UC2288 (UC2), or OHT alone, or in combinations. Populations of stained cells were gated into four quadrants: PI+/AV-FITC+ (upper right), PI-/AV-FITC+ (lower right), PI+/AV-FITC- (upper left), and PI-/AV-FITC- (lower left). **(B,C)** Luciferase reporter assays for the transcriptional activities of

endogenous ERα in MCF7-V (panel B) and T47D (panel C) cells. Cells were transiently transfected with the ERE-Luc reporter plasmids. Relative luciferase activities (RLU) were normalized to the activities of the internal transfection standard Renilla luciferase. Cells were treated with alpelisib (Alp), UC2288 (UC2), and OHT, tested alone or in the 3-drug combination (red bars). Luciferase activities of the vehicle-treated control groups were set to 1. **(D)** Luciferase reporter assays for exogenously expressed ERα with HEK 293T cells. Cells were transiently co-transfected with the ERE-Luc reporter and either empty vector pSG5 (EV) or pHEG0 (ERα). Drug treatments were as indicated and mentioned in panels B and C. Relative luciferase activities (RLU) were normalized to the activities of the internal transfection standard Renilla luciferase. Luciferase activities of the control cells transfected with EV and treated with DMSO were set to 1. **(E)** Immunoblots of the indicated proteins tested in total cell lysates of MCF7-V cell line. Cells were treated for 24 hours with either alpelisib (Alp), UC2288 (UC2), OHT, or talazoparib (Tala) tested alone, or the 3-drug combination (Alp + UC2 + OHT), or the 4-drug combination (Alp + UC2 + OHT + Tala). EGF (10 ng/ml) was added to cells 15 min before harvesting to induce MAPK signaling. GAPDH was used as an internal standard. **(F,G)** Luciferase reporter assays for the transcriptional activities of endogenous HIF1α activity in T47D (panel F) and MCF7/TamR (panel G) cells. Cells were transiently transfected with the HRE-Luc reporter plasmids. Relative luciferase activities were calculated and cells were treated as in panel B and C. For bar graphs, data are represented as means ± SD (n = 4 independent samples for panels B and C, and n = at least 3 for panels D, F, and G). Statistical significance between the groups were analyzed by one-way ANOVA and *p*-values ≤ 0.05 were considered statistically significant. Drug doses are listed in Table S1.

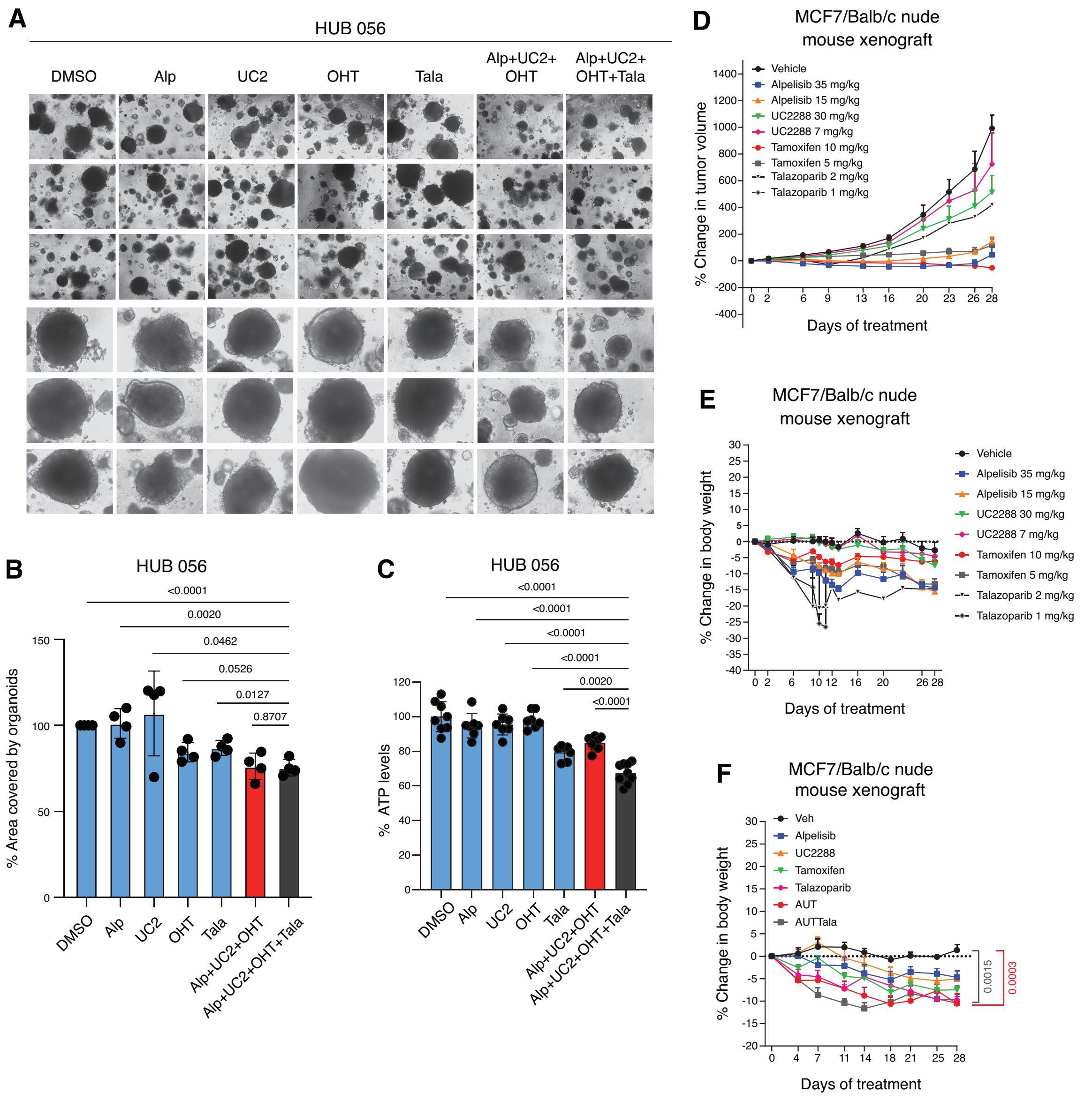

Fig. S10 Drug dose-responses with MCF7 xenografts. **(A)** Representative phase-contrast images of drug-treated organoids after 7 days of treatment. **(B)** Quantification of the area covered by organoids from multiple images of drug-treated organoids. **(C)** Bar graph of the % ATP levels of patient-derived mammary tumor organoid (model HUB 056) treated with alpelisib (Alp), UC2288 (UC2), OHT, and talazoparib (Tala) alone or in the combination of 3 drugs (Alp + UC2 + OHT) or 4 drugs (Alp + UC2 + OHT + Tala). ATP levels were tested after one week of treatment. The mean value of the DMSO control was set to 100%. Data are represented as means ± SD (n = at least 7 independent samples). For panels B and C, statistical significance between the groups were analyzed by unpaired student's t-test and *p*-values ≤ 0.05 were considered statistically significant. **(D,E)** Line graphs of the % change in tumor volume (panel A) and % change in body weight (panel B) of MCF7 xenografts in Balb/c nude mice treated as indicated and as in Table S1. Data points represent the means and error bars are SEMs. (n = 5 mice / group at the start of the treatments). In the group

treated with talazoparib 2 mg/kg, 1 mouse died and treatment was suspended for the rest of the group on day 9 due to severe toxicity (n = 0). Starting from day 13, 2 more mice died and 1 was euthanized due to poor conditions; only 1 mouse from this group remained alive till the end of the study but without being further treated as %BW loss was more than 15%. In the group treated with talazoparib 1 mg/kg, on day 6, treatment was suspended for one mouse (n = 4), on day 9, this mouse died and treatment was suspended for another one (n = 3), then on day 10, one more mouse died and treatment was suspended for the rest of the group (n = 0), and finally on day 12, the mice were euthanized due to severe poor conditions (n = 0). **(F)** Line graphs of the % change in body weight of MCF7 xenografts in Balb/c nude mice treated as indicated. Data points represent the means and error bars are SEMs. (n = 5 mice / group). For all panels, values on the first day of drug treatments (Day 0) were set to 0%. Differences in the mean values measured on day 28 of treatments were analyzed by unpaired student's t-test. *p*-values ≤ 0.05 were considered statistically significant. Drug doses are listed in Table S1.
